# Learning to Generate Sequences with Combination of Hebbian and Non-hebbian Plasticity in Recurrent Spiking Neural Networks

**DOI:** 10.3389/fnins.2017.00693

**Published:** 2017-12-12

**Authors:** Priyadarshini Panda, Kaushik Roy

**Affiliations:** Nanoelectronics Reserach Laboratory, Purdue Univerisity, School of Electrical and Computer Engineering, West Lafayette, IN, United States

**Keywords:** reservoir model, non-Hebbian learning, Hebbian plasticity, sequence generation, attractor dynamics, eigenvalue spectra

## Abstract

Synaptic Plasticity, the foundation for learning and memory formation in the human brain, manifests in various forms. Here, we combine the standard spike timing correlation based Hebbian plasticity with a non-Hebbian synaptic decay mechanism for training a recurrent spiking neural model to generate sequences. We show that inclusion of the adaptive decay of synaptic weights with standard STDP helps learn stable contextual dependencies between temporal sequences, while reducing the strong attractor states that emerge in recurrent models due to feedback loops. Furthermore, we show that the combined learning scheme suppresses the chaotic activity in the recurrent model substantially, thereby enhancing its' ability to generate sequences consistently even in the presence of perturbations.

## 1. Introduction

Learning to recognize, predict and generate spatio-temporal sequence of spikes is a hallmark of the nervous system. It is critical to the brain's ability for anticipating the next ring of a telephone or next action/movement of an athlete. Several neuroscience works have shown that such abilities can emerge from dynamically evolving patterns of neural activity generated in a recurrently connected neocortex (Klampfl and Maass, 2013; Laje and Buonomano, 2013; Zenke et al., 2015). Implementing a “*stable and plastic”* recurrent spiking neural network for learning and generating sequences with long-range structure or context in practical applications (such as text prediction, video description etc.), however, remains an open problem.

Here, we develop a reservoir spiking neural model with a biologically relevant architecture and unsupervised plasticity mechanisms that learns to generate sequences with complex spatio-temporal relations. Generally, reservoir networks, owing to their high level of recurrence, operate in a dynamic regime wherein stable input driven periodic activity and chaotic activity tend to coincide (Rajan, 2009; Rajan et al., 2010). We show that the chaotic activity can be reduced by effective tuning of connections within a reservoir with a multi-time scale learning rule and diverse plasticity mechanisms. This helps in the robust learning of stable correlated activity that is even resistant to noise. This further enables the reliable generation of learnt sequences over multiple trials lending our reservoir model a key feature characteristic of biological systems: *the ability to remember the previous state and return to the sequence being generated* in presence of perturbations.

Recent efforts in building functional spiking neural systems with Spike Timing Dependent Plasticity (STDP) for self-learning on practical recognition tasks have been mostly focused on feed-forward and hierarchical deep/shallow architectures (Masquelier and Thorpe, 2007; Diehl and Cook, 2015; Esser et al., 2016; Kheradpisheh et al., 2016; Panda and Roy, 2016) with minimal recurrent connections (for instance, recurrent inhibitory connections incorporated with such architectures for inducing competition and stabilizing neural activity in an unsupervised learning environment). Another body of work in the spiking domain encompasses “Reservoir or Liquid Computing” frameworks that attempt to capture the anatomy of the neocortex with substantial recurrent connectivity between groups of excitatory and inhibitory spiking neurons (Lukoševičius and Jaeger, 2009; Maass, 2010; Srinivasa and Cho, 2014). Jaeger (2001) proposed a similar randomly connected recurrent model, termed as echo state network, that uses analog sigmoidal neurons (instead of spiking) as fundamental computational units. In both liquid or echo state frameworks, the burden of training the recurrent connections is relaxed by fixing the connectivity within the reservoir and that from input to reservoir. Instead, an output layer of readout neurons is trained (generally in a supervised manner i.e., with class labels) to extract the information from the reservoir (Maass et al., 2002; Sussillo and Abbott, 2009). However, the fixed connectivity severely limits the ability of such frameworks to do general learning over varied applications. Such networks perform poorly as the number of possible patterns (or classes) as well as the temporal correlations (or context) between patterns increases.

It was shown recently that incorporation of synaptic plasticity within the reservoir can result in the emergence of long-term memory (Mongillo et al., 2003; Klampfl and Maass, 2013) that can help in learning and inferring relationships between inputs (Miller and Wingfield, 2010; Diehl and Cook, 2016). However, the main aim of those works were to build neural computing models that suitably elucidate the activity of the neocortex to better understand the mammalian brain. On the other hand, in this work, we take a more practical approach to engineer a recurrent spiking model capable of character-level recognition and word prediction. Essentially, given a dictionary of visual words, our spiking reservoir model learns to recognize each visual character (or alphabet) as well as the context/relation between subsequent characters of different words such that it can consistently generate the entire word. Note, in this work, we use the term “reservoir” in the context of a recurrent spiking neural network similar to that of a Liquid State Machine (LSM) (Maass, 2010) with spike-driven dynamics compatible with STDP.

Similar to Klampfl and Maass (2013) and Diehl and Cook (2016), we modify the synaptic weights of the connections from input to reservoir as well as the recurrent connections within the reservoir to develop the character-level language model. The learning is performed over different time scales. While the fast input to reservoir learning, operating in millisecond range, facilitates in recognition of individual characters, the slower learning of the recurrent connections, operating in the range of 100 milliseconds, within the reservoir enables the network to learn the context between the characters such that the learnt model can generate words by predicting one character at a time.

In addition, to reduce the strong/dominant attractor states that emerge from the strong feedback loops in the reservoir, we introduce a non-Hebbian adaptive weight decay mechanism in the learning rule. The decay in addition to STDP enables synaptic depression for hyperactive neurons in the reservoir that result from strong feedback dynamics instead of contextual dependencies. We show that the combined effects of Hebbian and non-Hebbian plasticity mechanisms results in a *stable-plastic* recurrent SNN capable of generating sequences reliably. We also justify the effectiveness of the combined plasticity scheme by analyzing the eigen value spectra of the synaptic connections of the reservoir before and after learning. As shown in later sections, this theoretical analysis provides a key insight about the inclusion of non-Hebbian decay to reduce chaotic dynamics within a reservoir.

## 2. Materials and methods

### 2.1. Reservoir model: framework and implementation

#### 2.1.1. Network architecture

**Figure 1:**
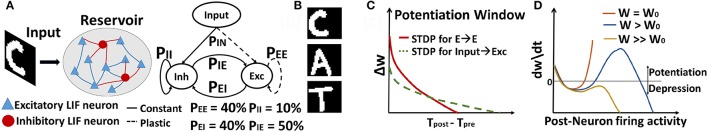
**(A)** General topology of Recurrent SNN used for sequence learning and prediction **(B)** Sample image of dictionary of visual words **(C)** STDP Potentiation window for Hebbian Phase learning of In→Exc & E→E reservoir connections over diverse time scales **(D)** Synaptic changes with the combined Hebbian/non-Hebbian Plasticity as a function of rate of post-synaptic neuron that prevents strong attractor dynamics by regulating the over-potentiation of synapses. Note, **(C,D)** are cartoons (that do not depict empirical data) to show the behavior of STDP based weight change for slow/steep learning in **(C)** and effect of inclusion of non-Hebbian decay on the hyperactive neuronal weights in **(D)**.

The general architecture we consider is a 2-layered network as shown in Figure 1A. The topology consists of an input layer connected to a reservoir of *N* Leaky-Integrate-and-Fire (LIF) neurons (Diehl and Cook, 2015), with a connection probability *P*_*IN*_ of 30%. The input layer contains the pixel image data (with one neuron per pixel), corresponding to the visual words or characters in the dictionary. Of the total *N* neurons in the reservoir, 80% are excitatory and 20% are inhibitory, in accordance with the ratio observed in the mammalian cortex (Wehr and Zador, 2003). The reservoir is composed of all possible combinations of recurrent connections, depending upon the pre- and post-neuron type at each synapse: E→E, E→I, I→E, I→I, where E (I) denote excitatory (inhibitory) neurons. These recurrent connections are set randomly with a relatively sparse connection probability of *P*_*EE,EI,IE,II*_. Here, all connections going to excitatory neurons (i.e., input to reservoir excitatory neurons and E→E connections within the reservoir) are plastic, while all other connections maintain their initial random values.

Note that the synaptic weight modification of the plastic connections shown in Figure 1A helps in learning the rich temporal information present in the input data and also enables the understanding of contextual dependencies (for learning a word from individual characters) that span over multiple time steps. Another interesting property of our model is that it does not contain a readout layer that is generally present across all conventional reservoir spiking models (Legenstein and Maass, 2007; Maass, 2010; Srinivasa and Cho, 2014) for sequence recognition. The synaptic plasticity from input to reservoir, specifically, helps in learning the generalized representations of the input patterns (i.e., images of characters in this case). This in turn enables us to perform unsupervised inference without a readout layer of output neurons.

In Figure 1A, the connection probabilities and the Excitatory(E)/Inhibitory(I) neuron ratio in the reservoir are chosen such that we have a balanced contribution of E/I synaptic currents that contribute to the spontaneous activity of the reservoir (Wehr and Zador, 2003). Generally, the number of E/I neurons in the reservoir maintain a 4:1 ratio as observed in the neocortex. Since E>I, inhibitory connections must be larger than excitatory connections (i.e., *P*_*EE*_ < *P*_*EI*_ × *P*_*IE*_). The connection probabilities are usually set randomly while ensuring that E/I balance is maintained. However, a simple excitation of the reservoir with noisy Poisson inputs and observing the trajectory (or neuronal firing activity from the reservoir) generally helps to choose a probability range. For such random noisy inputs, the reservoir will be chaotic and should ideally show irregular trajectory with varying firing activity for every stimulation. However, if excitation dominates (*P*_*EE*_ > *P*_*EI*_ × *P*_*IE*_), then, there will be a mean positive drift of the neuronal membrane potential toward the threshold, resulting in a fixed trajectory (or regular firing activity). In contrast, if excitation and inhibition balance each other, the membrane potential will follow a random trajectory resulting in a trajectory with Poisson statistics as expected. This happens when *P*_*EE*_ < *P*_*EI*_ × *P*_*IE*_. While it might seem that a lot of synaptic fine-tuning is required, on the network level, such an E/I balance can arise dynamically if two conditions are met. First, connections must be sparse and random as shown in Van Vreeswijk and Sompolinsky (1996). Second, connections for inhibitory must be greater than excitatory. We follow both these rules in addition to observing the reservoir trajectory for random noise triggering while initializing our model. *P*_*IN*_ (30%) is set such that a minimal firing rate of 15–20 Hz is observed from the reservoir for a ~45 Hz input that will allow significant synaptic learning within the STDP timing window of simulation.

#### 2.1.2. Synaptic plasticity and homeostasis

The synapses connecting the input to excitatory reservoir neurons (In→Exc) and the E→E connections within the reservoir are trained using a combination of Hebbian STDP and non-Hebbian Heterosynaptic plasticity that prevents strong feedback loops in the recurrent connections. STDP is a widely used weight update rule to accomplish unsupervised learning in SNNs. The weights of the synaptic connections are strengthened or weakened based on the time interval elapsed between pre- and post-synaptic spikes. We adopt different forms of weight dependent STDP rule to compute the weight updates. To implement the non-Hebbian plasticity, we introduce an adaptive decay mechanism (Chen et al., 2013; Chistiakova et al., 2014; Panda et al., 2017) (that only depends on the state of the post-synaptic neuron) in the weight update rule.

In addition to synaptic plasticity, we employ a homeostatic membrane threshold mechanism (Zhang and Linden, 2003), for the excitatory neurons in the reservoir that regulates the firing threshold to prevent a neuron from being hyperactive and dominating the overall response. Specifically, each excitatory neuron's membrane threshold is not only determined by υ_*thresh*_ but by υ_*thresh*_+θ, where θ is increased each time when the neuron fires and then decays exponentially (Querlioz et al., 2013). It is worth mentioning here that the interplay between homeostatic threshold and combined Hebbian/non-Hebbian plasticity results in a stable and plastic network with a balance between excitatory and inhibitory currents at each neuron in the reservoir.

In Klampfl and Maass (2013), Srinivasa and Cho (2014) and Diehl and Cook (2016), the authors have used inhibitory STDP to induce homeostasis or balance the activity of the excitatory neurons by modifying the synaptic weights of I→E recurrent connections. This is in stark contrast to our model where only the E→E connections within the reservoir are plastic. We note that among all the recurrent connections, E→E help learn the context between subsequent patterns while reinforcing patterns with similar statistics. The remaining connections mainly contribute to fostering competition (E→I) among different excitatory neurons to learn different patterns while maintaining a balanced and asynchronous firing activity (I→I, I→E) in the reservoir. Since the main aim of our model is to learn the underlying representations of visual inputs and understand the correlation between subsequent patterns, we can achieve this simply with E→E plasticity, while maintaining an optimum fixed and sparse connectivity across remaining connections as specified in Figure 1A.

### 2.2. Sequence learning with the proposed reservoir model

Given a visual word “CAT” composed of individual characters as shown in Figure 1B, our model processes each character individually to learn the representations of each character with Input to Excitatory reservoir (In→Exc) plasticity. Simultaneously, the plasticity among the E→E connections within the reservoir should be such that the network learns that “CAT” is one entity with a sequential correlation (i.e., “C” is followed by “A” followed by “T”). During testing, when the model is presented with a test image “C”, the network should recognize the character and output the next most probable character from the entity it has learnt previously. In order to perform such sequence generation, we conduct the learning in two phases as described below.

#### 2.2.1. Hebbian phase

In this phase, we modify the synaptic weights of both In→Exc and E→E connections with different forms of STDP. For In→Exc plasticity that helps in learning the underlying representations of the individual images/characters, we perform the weight updates using the power law weight dependent STDP rule (Querlioz et al., 2013; Diehl and Cook, 2015), illustrated in Figure 1C. To improve simulation speed, the weight dynamics are computed using synaptic traces as proposed in Morrison et al. (2007). Besides the synaptic weight, each plastic synapse keeps track of the pre-synaptic (or post-synaptic) trace, *x*_*pre*_ (or *x*_*post*_). Each time, a pre (or post) neuron fires, the corresponding trace is increased by 1, otherwise *x*_*pre*_ (or *x*_*post*_) decays exponentially. When a post-synaptic neuron fires a spike, the weight change Δ*w* is calculated as

(1) $$\begin{array}{l} \Delta w_{In\to Exc}=\eta [(x_{pre}-offset){\left(w_{max}-w\right)}^{\mu })] \end{array}$$

where η (0.05) is the learning rate, *w*_*max*_ (1.0) is the maximum constraint imposed on the synaptic weight, *x*_*pre*_ is the pre-synaptic trace value that exponentially decays with τ_*pre*_ = 30 *ms* and *w* is the current weight value. The synaptic strength is increased by *w* = *w* + Δ*w*_*In*→*Exc*_ if a pre-neuron subsequently causes the connected post-neuron to fire which signifies a strong causal relationship. On the other hand, the synaptic strength is decreased for larger spike time differences as determined by the *offset*. This training enables the excitatory neurons in the reservoir (connected to the input) to encode a generic representation of an image pattern in their corresponding In→Exc weights.

Concurrent to the above training, we simultaneously modify the E→E connections within the reservoir with steeper STDP learning as shown in Figure 1C. For the E→E weight updates, we use a modified version of the exponential weight-dependent STDP rule (Pfister and Gerstner, 2006; Diehl and Cook, 2015). The synaptic weight updates based upon the arrival of pre- and post-synaptic spikes are again calculated using synaptic traces as follows:

(2) $$\begin{array}{l} \Delta w=-\eta_{1}[x_{post}w^{\mu }](when\;pre-neuron\;fires)\\ \Delta w=\eta_{2}[x_{post}x_{pre^{\prime }}{\left(w_{max}-w\right)}^{\mu })](when\;post-neuron\;fires) \end{array}$$

where η_1,2_ is the learning rate (0.002, 0.01) and *w*_*max*_ value is 0.5. Similar to above learning, the weights are potentiated or depressed based on the spike timing correlation. However, with slower learning rate and smaller time constants of decay for the pre-/post-synaptic traces ($x_{pre^{\prime }}/x_{post}$), $\tau_{pre^{\prime },post}=10,20\text{ ms}$, significant synaptic weight updates are carried out for really small spiking differences between pre- and post- neurons in this case. This slow learning is desirable as the E→E connections must encode the correlation between the individual images. Hence, the E→E connections should get updated only when both the pre- and post- excitatory neuron in the reservoir have spiked very closely and over longer periods of time. The synaptic traces will increase more and cause meaningful weight updates for such stronger causal relationship. Please note that all the pre- and post-synaptic traces in the above learning rules are disjoint and calculated separately. Furthermore, the weight dependence terms in the above equations prevents abrupt or fast change in weight values. Note, μ value is 0.9 for both Equations 1, 2.

#### 2.2.2. Non-Hebbian phase

In addition to the exponential STDP learning, the E→E connections further undergo a decay toward a baseline value (*w*_0_ = 0.2) to prevent the reservoir plasticity to cause strong feedback loops in the network thereby curtailing the emergence of dominant attractor dynamics. Such strong attractor states cause the network dynamics to converge to the same state for different input sequences, drastically degrading the inference capability of the network. For instance, if the network learns the words “CAT”, “COT” wherein the recurrent connections are strongly correlated for excitatory neurons representing “C”, “O”, and “T” then, the network will only output “O” when presented with “C” during testing. Ideally, we would like our Reservoir Model to give all possible sequences or words for a given input.

The decay of E→E synaptic weights is performed at every simulation time step as

(3) $$\begin{array}{l} \frac{dw}{dt}=-\gamma \left(t\right)\left(w-w_{0}\right) \end{array}$$

where γ(*t*) is the decay rate that is a time dependent quantity proportional to the squared post-synaptic trace value (${\left(x_{post}\right)}^{2}$ from Equation 2) and the homeostatic membrane threshold value (υ_*thresh*_ + θ) at a given time instant. The direction of change depends on the present value *w* of the synaptic weight in relation to the baseline value *w*_0_. If the post-synaptic neuron fires more due to the strong feedback loops, the decay rate proportionately increases weakening the E→E connections thereby reducing the influence of very strong attractor states on the network dynamics.

In some situations, dominant attractor states may also arise due to disproportionate input sequences. For instance, if the network learns the words “CAT”/“CRAFT”, “CRAFT” on account of being a longer sequence will induce stronger correlation between the neurons representing the underlying characters thereby creating an imbalanced recurrent model. The homeostatic membrane threshold of an excitatory neuron is representative of the length of the input pattern. For a longer sequence, E→E connections are reinforced more with the exponential rule (Equation 2) such that the neuron learns the correlation well. The reinforcement in turns causes the neurons to spike more thereby increasing their membrane threshold. Higher membrane threshold correspondingly increases the decay rate, thus, preventing the length of input sequences from causing strong attractor states. Please note that this decay mechanism is non-Hebbian (does not involve pre, post spike timing correlation) and bears resemblance to the heterosynaptic plasticity observed in the mammalian brain that prevents runaway synaptic dynamics and stabilizes the distribution of synaptic weights (Chen et al., 2013; Chistiakova et al., 2014).

The rate of change of weight with our combined plasticity scheme is illustrated in Figure 1D. The adaptive decay mechanism helps in un-learning the weights of neurons that become hyperactive due to strong feedback loops. For synaptic weights that are larger than the baseline value *w*_0_, it allows synaptic depression of E→E connections even for high post-neuronal firing activity that is not possible with lone exponential STDP learning. In addition to reducing the strong attractor states, the synaptic decay acts a memory consolidation mechanism wherein certain connections that are relevant for the reliable generation of the sequence are selectively potentiated while depressing recurrent connections that lead to undesirable correlation. As mentioned earlier, both In→Exc and E→E connections are updated simultaneously with the above mentioned rules (Equations 1–3). The In→Exc learning is done at each time step based upon pre/post firing (order of ms). In contrast, the Exc→Exc weight updates occur slowly over multiple time steps due to the slower STDP learning. This concerted interplay of the multiple plasticity mechanisms (both Hebbian and non-Hebbian) leads to a stable reservoir that avoids strong/dominant attractor states and reliably generates all possible answers for different sequence of words learnt.

In our simulations, each individual character of the word sequence (that is a 28 × 28 pixel image of “A to Z”) is presented to the reservoir as Poisson spike trains (with firing rates proportional to the intensity of the pixels) for 350 ms with a simulation time step of 0.5 ms. For instance, while learning “CAT”, each individual character (“C”, “A”, and “T”) is shown individually and sequentially for 350 ms each. Then, before presenting the next sequence/word (for instance, “COT” or a different representation of “CAT”), there is a 300 ms phase without any inputs to allow all variables of all neurons to decay to their resting values (except the adaptive membrane threshold). This sequential presentation of the characters (without resetting the membrane potential of the neurons until the entire sequence or word is shown) helps the E→E connections to learn the correlation between them.

Note, the Supplementary Material (at the end of the paper) contains additional details regarding the neuron/synapse model, input encoding method and the training/assignment/inference methodology. Please refer it to get further insights about the intrinsic parameter values.

## 3. Results

The proposed reservoir model and learning was implemented in BRIAN (Goodman and Brette, 2008). We created a dictionary of visual words (samples shown in **Figure 3A**) from the handwritten characters in Char74K dataset (de Campos et al., 2009) that were used to train and test our reservoir model for sequence learning.

**Figure 2:**
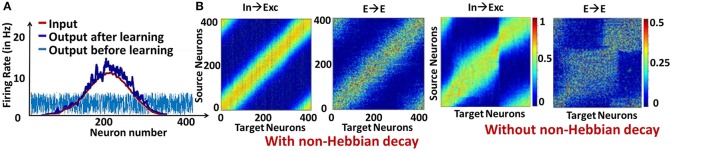
**(A)** Response pattern of reservoir neurons for Gaussian Input profile (average: 5 Hz) before and after learning **(B)** Visualization of the weight matrices between Input→Exc and E→E reservoir connections learnt with and without non-Hebbian decay.

We first show the effectiveness of the combined plasticity mechanism in reducing the strong/dominant attractor dynamics that emerge in a recurrent spiking neural network. We simulated a reservoir model of 400 excitatory, 100 inhibitory LIF neurons with an input layer composed of 400 Poisson Spike Generators that gives the input a Gaussian shaped firing rate profile. In this case, learning the In→Exc and E→E connections as described earlier (along with non-Hebbian decay) causes the reservoir neurons' spiking response (that fired randomly before training) to match the firing rate profile of the input as shown in Figure 2A. Correspondingly, the weight matrices for the In→Exc connections and E→E connections within the reservoir form a diagonal structure representative of the bell-shaped Gaussian distribution of the input patterns over the reservoir neurons, as illustrated in Figure 2B. In contrast, the weight values accumulate in certain regions causing strong attractor dynamics in the reservoir because of the feedback loops when the non-Hebbian decay based plasticity is not incorporated in the E→E synaptic learning as shown in Figure 2B. Such weight crowding is caused by the Hebbian nature of lone STDP learning. Since STDP causes reinforcement of correlated activity, the feedback loops between sub-groups of neurons that are strongly interconnected due to the recurrent dynamics of the reservoir will over-potentiate the E→E connections, further causing them to be overly active. As a result, the weights get crowded instead of having a homogeneous distribution. Inclusion of non-Hebbian decay in the learning mechanism helps in decreasing the activity of such sub-groups of neurons by enabling synaptic depression even at high post-synaptic firing rates as seen earlier (refer to Figure 1D).

**Figure 3:**
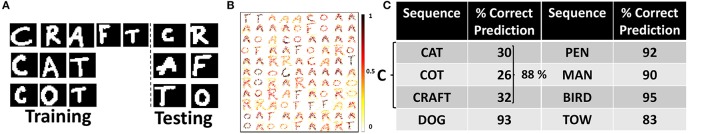
**(A)** Sample training and testing images of visual words **(B)** Representations encoded by In→Exc connections of 100 excitatory neurons in a 200-neuron reservoir. The color intensity of the patterns are representative of the value of synaptic weights (after training) with lowest intensity (white) corresponding to a weight value of “0” and highest intensity (black) corresponding to “1”. **(C)** Percentage of correct predictions made by the 400-neuron reservoir for different sequences during testing. The prediction accuracy is averaged across 100 trials of different presentations of the test input characters: “C”, “D”, “P”, “M”, “B”, and “T”.

Now, we discuss the recognition and generation of words from visual characters. We simulated a reservoir model of 200 neurons (160 excitatory, 40 inhibitory) to recognize a dictionary of words: “CAT”, “CRAFT”, “COT”. Please note, the words selected are such that some sequences have more common underlying characters (such as CAT, COT) than others. To validate that our model is effective and can generate all possible learnt sequences consistently without any bias toward a particular sequence, we use the above selection. For training, we use 200 different representations for each word composed of dissimilar characters (i.e., total 600 words for training). For testing, we used 100 distinct representations of each character. Figure 3A shows the sample training and testing images used. After training, the In→Exc connections that are essentially responsible for recognizing the individual characters of the word encode the generalized representations of the individual characters as shown in Figure 3B. Individual characters get associated with different excitatory neurons in the reservoir that fire steadily when presented with an input similar to the pattern it has learnt.

During testing, when an input pattern is shown, several excitatory neurons assigned to different characters may potentially fire. For example, for a test image of “C”, a group of neurons associated to “D” may also fire. The spiking/firing rates for different groups will however differ (say spiking rate of “C” > “D”). However, since we learn the E→E connections in the reservoir while presenting the individual characters sequentially (without resetting the membrane potentials) during training, the average firing rates of the neurons in the reservoir interestingly follow a particular sequence. For a test image of “C”, the top-2 average spiking activity are observed for neurons associated with “C”, “A”. Further, the difference between the top-2 spiking activities is quite low (an average of ~ 3-4). Similarly, based on the second highest spiking activity, we input the next character (i.e., “A” in this case) to which the top-2 spiking activity recorded are for neurons associated with “A”, “T”. Next, when we input “T”, while the highest spiking activity is observed for “T”, the second highest activity is quite random (associated with different neurons for varying test presentations) with an average spiking difference that is >10. The large spiking difference in the top-2 activity indicates that the last character of the sequence has been recognized by the reservoir for a given test trial and no further inputs are then provided to the reservoir. Please note, similar to training, the membrane potentials of the neurons in the reservoir are not reset in a test trial until the entire sequence/word is generated or a large spiking difference in top-2 activity is observed. We observe such consistent sequential generation of top-2 spiking activity across all test trials. As the dictionary contains 3 words starting with “C”, the second highest spiking activity alters between “R”, “A”, and “O” for different trials. This difference arises due to the randomness in the Poisson distribution of inputs. In fact, for 100 trials, when the reservoir model was presented with test input “C” the network yielded a correct word from the dictionary 85 times with “CRAFT”, “CAT”, and “COT” generated 34, 23, 28 times respectively. In the remaining 15 trials, some garbage words such as “CAFT”, “CRT” etc. were generated.

Next, we simulated a reservoir model of 400 neurons (320 excitatory, 80 inhibitory) to learn a larger dictionary with: “CAT”, “COT”, “CRAFT”, “DOG”, “PET”, “MAN”, “BIRD”, and “TOW”. Figure 3C shows the number of times a correct sequence is generated by the reservoir across 100 different presentations of the first character of every word: “C”, “D”, “M”, “B”, “T”. The average accuracy of the reservoir is 91.2% wherein the sequence generation was more accurate for “PET”, “DOG”, and “BIRD” that share less characters with the remaining words of the dictionary. Minimum accuracy is observed for “TOW” since presentation of “T” in most cases yielded a large difference in the top-2 spiking activity of the neurons. This can be attributed to the fact that “T” being the last character for most words limits the neuron learning a “T” to develop contextual dependencies toward other neurons. Few garbage words generated by the reservoir include “COW”, “CRAT”, “DOT”, “BRD”, “PT”, “MAT”, and “BRAT” among others. Of the garbage words, it is quite remarkable to see that some are actual English words that the reservoir generates not having seen them earlier. Another noteworthy observation is that most garbage words end with “W”, “T”, and “D”, that are essentially the last characters of the words in the dictionary. Please note that in the words present in the dictionary, repetition among characters is not present (for instance, “SEEN” or “RALLY”). In such cases, our reservoir model generates “SEN”, “RALY” instead of the correct words. Since the generation of sequences in our reservoir model is based on top-2 spiking activity, when “E” is presented to the reservoir, the top-2 highest spiking activity with minor difference is observed for neurons associated with “E”, “N”. We cannot identify such repetition among characters with this scheme.

**Figure 4:**
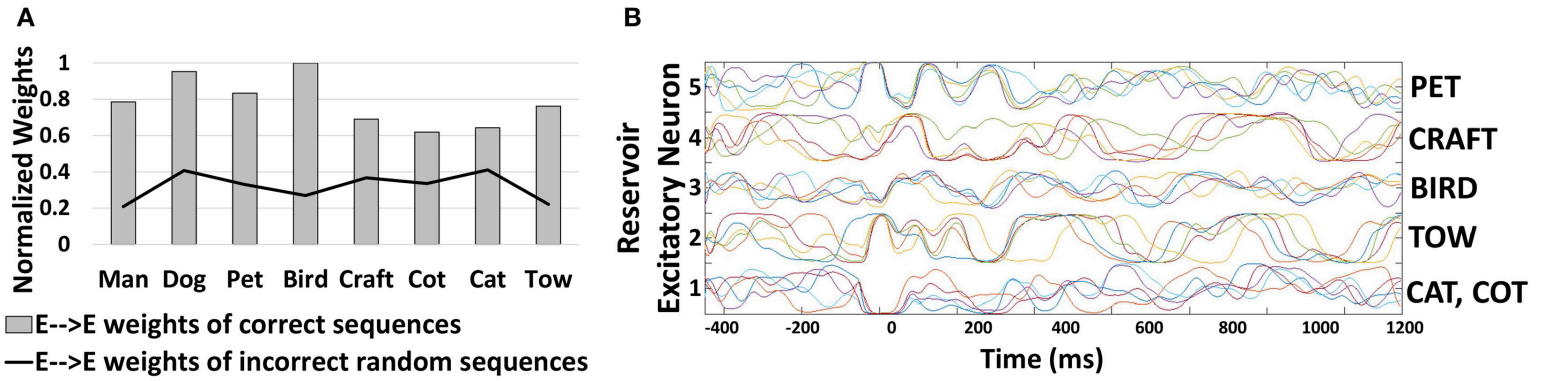
**(A)** Normalized average value of trained weights of E→E reservoir connections corresponding to different correct/incorrect sequences predicted by the 400-neuron reservoir model. All weight values are normalized with respect to the highest average value (0.42) recorded for the sequence “BIRD” in this case. **(B)** Firing rates/Trajectories of 5 excitatory neurons in the 400-neuron reservoir encoding different characters. Different color coding of trajectories specify different trials.

In order to demonstrate the effectiveness of our combined learning scheme for generating sequences, we recorded the average synaptic weights of the E→E connections (that encode the correlation) learnt among the excitatory neurons associated with different characters. Figure 4A shows that the average value of the recurrent connections learnt by the 400-neuron reservoir model described above, differs across different words with minimal variation. In fact, for words with similarities (such as “CAT”, “COT”, and “CRAFT”) the weights have almost similar values. The slight variation across the weight values is indicative of the fact that the reservoir model (after learning) has reduced chaotic activity. Generally, all recurrent networks exhibit some chaotic activity owing to the feedback connections that, if not controlled, can cause abrupt changes in neuronal activity leading to a severe degradation in their inference ability. The reduction in chaotic states enables our model to produce stable recurrent activity while generating a particular sequence. This further establishes the suitability of our combined learning scheme for reservoir plasticity. Additionally, the strength of the connections between neurons associated with random sequences (that are not present in the dictionary) such as “DR”, “POM”, “TMO”, “CG” etc. are very low (2.46x lesser than the mean of all connections generating relevant sequences). This result illustrates that the proposed reservoir model learns stably to form and retrieve relevant sequences.

To supplement the above result of reduction in chaotic states, we plot the trajectories (i.e., firing rates of neurons as time evolves) of 5 excitatory neurons in the 400-neuron reservoir that encode the characters (“C”, “B”, “P”, and “T”) across 5 test trials as shown in Figure 4B. It is clearly seen that the activity of each neuron prediciting a particular word follows a stable trajectory with slight variation across different trials for different sequences. When the network is presented with any input (say “P” corresponding to Unit 5), initially, the trajectories vary across different trials until they converge around time t = 0 ms, where the highest spiking activity is observed for the given character in that particular neuron. Based on the second highest activity, when the next character is presented to the network, the neuronal activity of the neuron across different trials varies as expected (due to the randomness in the input distribution as well as the chaotic activity). However, the trajectories tend to converge toward one another with time implying more correlated activity. In fact, sequences that have less commonality with other words (“PET” and “BIRD”) have more synchronized trajectories. Thus, we can infer that the contextual dependencies developed with the modification of E→E connections has a stabilizing effect on the chaotic states of the reservoir. While the existence of chaotic states help a neuron differentiate between sequences that have more similarities (such as “CAT”, “COT”), it does not have an overwhelming effect disrupting its inference capability thereby enabling our model to operate at the *edge of chaos*.

**Figure 5:**
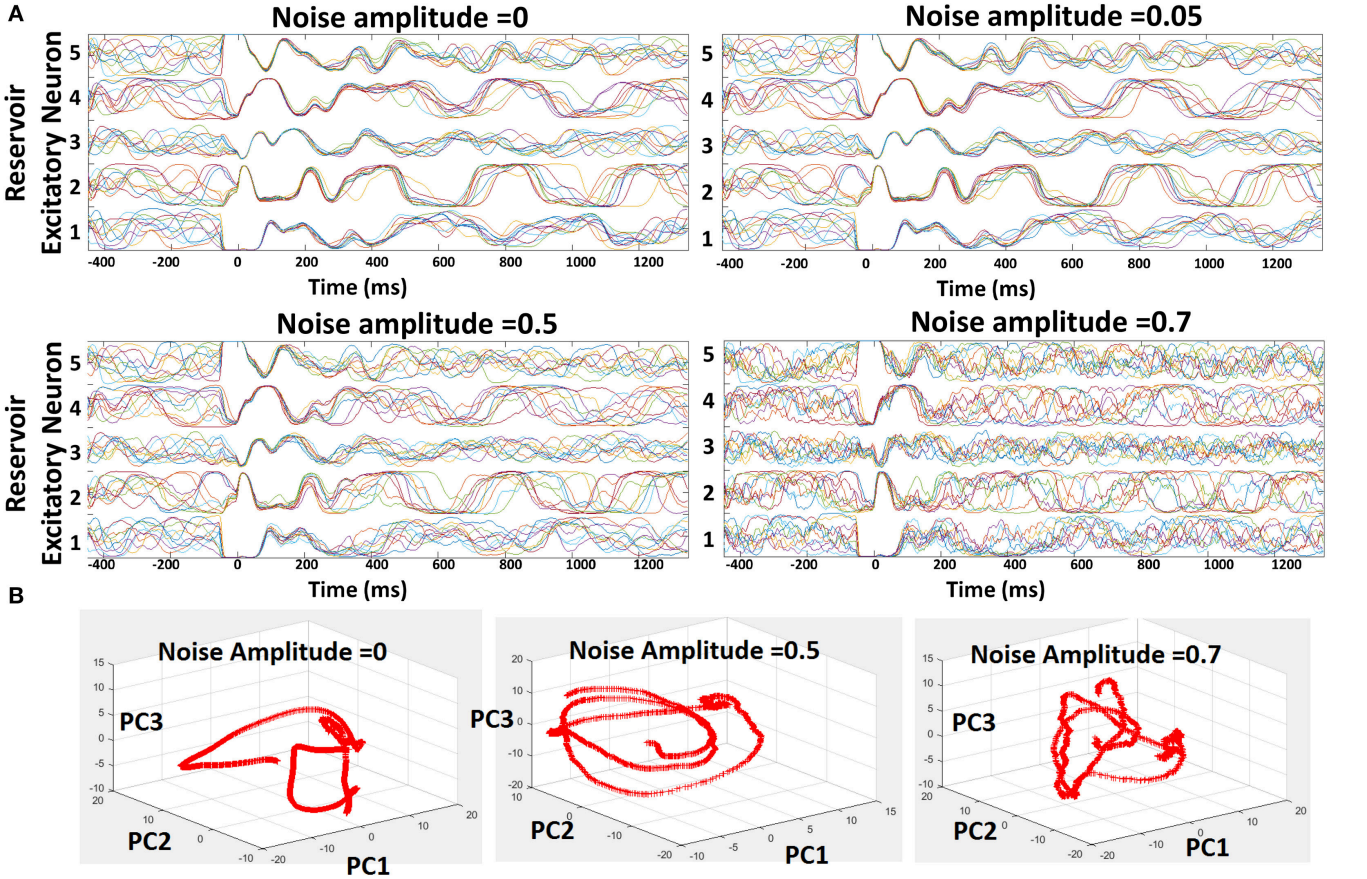
Robustness of the reservoir spiking model learnt with combined Hebbian/non-Hebbian plasticity against noise. **(A)** Trajectories shown for 5 different excitatory neuron of the 400-neuron reservoir model for varying noise activity across 10 different test trials. **(B)** Projections of the spontaneous activity of the excitatory neurons in the reservoir onto Principal Component Vectors 1, 2, 3 plotted against each other in a 3-D manner.

Suitably learnt reservoir models generally operate with co-existing chaotic states and stable trajectories as shown above. However, external noise can induce more chaos in the reservoir that will overwhelm the stable patterns of activity due to the inevitable feedback loops among recurrent connections. Here, we discuss the susceptibility of our reservoir model (and hence our learning scheme) to external noise. To conduct the noise analysis, we introduced random Gaussian noise along with the standard Poisson inputs to the 400-neuron reservoir model during testing. The injection of noise alters the net post-synaptic current received by the reservoir neurons (*I*_*post*_ = Σ_*i*_[*W*_*i*_*Input*_*i*_ + *N*_0_*randn*(*i*)], where *N*_0_ is defined as the noise amplitude) thereby affecting the overall firing rate (or trajectory). In order to prevent the variation in trajectory caused due to the randomness in the Poisson inputs, we fixed the distribution for a given input/chararcter across different trials. Figure 5A shows the trajectories of five different recurrent units for varying levels of noise (with different *N*_0_) for 10 different test trials. Since the input distribution is constant, the neuronal trajectory during the presentation of the first character in each trial (from t = 0 to t = 350 ms) remains almost equivalent in all cases. As time evolves, we input the next character predicted by the reservoir based on the top-2 spiking activity that leads to a different trajectory for each neuron, representative of the chaotic states. As *N*_0_ increases, we observe a steady increase in the variation of the trajectories which in turn degrades the capability of each neuron to predict correctly. However, only for noise levels with *N*_0_ ≥ 0.7, the reservoir yields diverse trajectories. For moderate noise (with *N*_0_ ≤ 0.5), our model exhibits high robustness with negligible degradation in prediction capability. In addition to visualizing the trajectories, we also plotted the Principal Components (PCs) of the firing activity of the excitatory neurons in the reservoir against each other, as shown in Figure 5B, for varying levels of noise. Generally, the first 10 principal components account for significant portion of variance in the chaotic spontaneous activity of the reservoir (Rajan, 2009; Rajan et al., 2010). Hence, we plotted the projection of the activity of the neurons onto PC vectors 1, 2, 3, and visualized them in a 3D space. It is clearly seen that the addition of external noise of varying amplitude changes the trajectory themselves. This shows that the neuronal activity or trajectories are not simply variable in time due to added noise. Instead, there is a drastic transformation of the trajectory itself that probably kicks the reservoir out of a stable state into a chaotic one limiting its' prediction capability.

**Figure 6:**
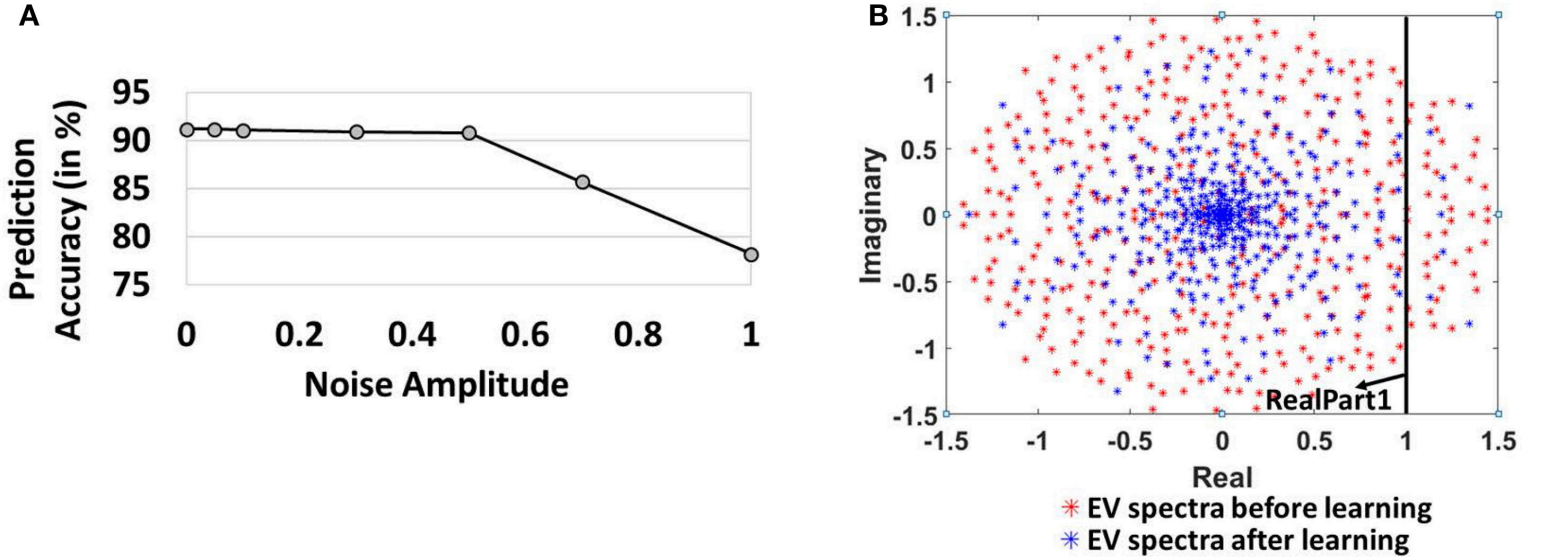
**(A)** Variation of Prediction accuracy with noise amplitude **(B)** Evolution of spectrum of eigen values of the reservoir synaptic connections (includes *E* → *E, E* → *I, I* → *E, I* → *I*) in the complex plane before and after learning.

Figure 6A also shows the prediction accuracy of the 400-neuron reservoir model for varying noise levels. For noise amplitude of 0.5, the accuracy observed is 90.8% (0.4% lower than the model without noise). Our model's insensitivity to adequate levels of noise further validates the efficacy of the combined Hebbian/non-Hebbian plasticity learning in reducing the chaotic states within the reservoir model. The reduction in chaotic dynamics during the training phase allows the network to look back in history to formulate the predictions correctly even in presence of external noise. The accuracy levels, however, degrade steeply with increasing levels of noise (*N*_0_ beyond 0.5) as the chaotic activity (due to the recurrent feedback loops) starts overwhelming the locally stable reservoir activity. For a noise amplitude of 1.0, the accuracy observed is 78.2%.

To further elucidate the effectiveness of Hebbian/non-Hebbian plasticity in reducing chaos, we probed into random matrix theory that gives a powerful understanding of the complex emergent behavior of large networks of neurons, such as the reservoir, with random recurrent connections. Diagonalization of the synaptic weight matrix of the reservoir connections yields equal number of modes as the number of neurons in the reservoir (Rajan and Abbott, 2006). Each mode can be labeled by a complex numbered eigenvalue, whose real part corresponds to the decay rate of an associated mode and imaginary part is proportional to the frequency of the pattern. Activation of any one of these complex modes results in a network that exhibits spontaneous oscillations at the corresponding frequency (Rajan, 2009). The activation of multiple such modes results in complex dynamics due to a superposition of individual frequencies in a highly nonlinear manner resulting in chaotic dynamics/persistent reservoir activity as observed earlier in Figures 4B, 5. We analyzed the EigenValue (EV) spectra of the synaptic weights of the reservoir before and after learning. The EV of all the connections (includes *E* → *E, E* → *I, I* → *E, I* → *I*) in the reservoir initially (drawn from a random distribution) are distributed uniformly in a circle in the complex plane in accordance with Girko's circle law (Girko, 1985). If the real part of a complex eigenvalue exceeds 1, the activated mode leads to oscillatory behavior contributing to the overall chaotic dynamics of the reservoir. From Figure 6B, we observe that before learning, the eigenvalues of the 400-neuron reservoir have a larger radius with more values > RealPart1 implying more activated modes characteristic of chaos (Rajan and Abbott, 2006). However, as learning progresses, the EV spectral circle shrinks to a non-uniform distribution with a high density of values toward the center and few modes > RealPart1. The dense center EVs are fixed points that are non-chaotic and persistent. In fact, the changing shape of EV spectrum with training of *E* → *E*, computationally corresponds to learning. The gradual movement of EV spectra from chaotic to fixed points establishes the stabilizing effect of learning the E→E reservoir connections with our combined plasticity scheme.

## 4. Discussion

We presented an unsupervised recurrent spiking neural model learnt with different forms of plasticity for reliable generation of sequences even in presence of perturbations. We incorporated a non-Hebbian decay mechanism, inspired from the Heterosynaptic plasticity observed in mammalian brain, with standard STDP learning while evolving the recurrent connections within the reservoir. The combined learning scheme suppressed the chaotic activity of the network while reducing the dominant attractor states and substantially enhanced the inference ability of the reservoir.

Our results indicate that the mutual action of the Hebbian/non-Hebbian plasticity enables the formation of locally stable trajectories in the reservoir that works in symphony with the reduced chaotic states to produce different sequences. While we demonstrate the efficacy of our model for fairly simple character-level prediction of visual words, we believe that the general functional properties of our combined plasticity learning can be extended to larger recurrent models for more complex spatio-temporal pattern recognition (such as action recognition, video analysis etc.). Investigation of the learning rule on other recurrent architectures is a promising direction of future research. However, large-scale networks, with a larger number of recurrent connections, are more vulnerable to chaotic dynamics, that would require a larger number of training examples for convergence. This will affect the training complexity of the reservoir. In such cases, the decay rate as well as the learning rate (in Hebbian phase learning) has to be varied suitably to avoid the formation of strong feedback dynamics while maintaining reasonable training time. Also, the inference ability of the reservoir is a strong function of the synaptic connecticity (or size of the reservoir). To avoid escalating the reservoir size for more complex problems, the learning rules can further be optimized. For instance, varying the learning rate as training progresses or a mechanism that adapts the decay rate (in the non-Hebbian phase) depending upon the extent/intensity of attractor states formed, might further enhance the inference ability of a reservoir.

It is worth mentioning that there has been previous effort on combining different plasticity schemes with reservoir models. In Lazar et al. (2009), the authors combine STDP, synaptic scaling of *E* → *E* connections and homeostasis to engineer a plastic reservoir model that performs better than static networks on simple tasks. While our paper complements Lazar et al. (2009), the inclusion of non-Hebbian decay gives an entirely new perspective toward learning in recurrent networks with reduced chaos and regular trajectories even in presence of noise. Furthermore, this work deals with more difficult visual character prediction while entailing a detailed analysis of the contribution of the Hebbian/non-Hebbian Plasticity scheme for stable memory states. Finally, we would like to mention the recent work Thiele et al. (2017) that deals with reducing the dominant attractor states developed in a recurrent SNN (different from our model) by un-learning the strong feedback connections after training. In contrast, the combined plasticity scheme proposed in this paper can serve as a general learning methodology that *cohesively reduces the synaptic weights of strong correlations during training* for more robust and noise-resilient recurrent SNN implementations.

## Author contributions

PP and KR conceived the study, PP conducted the experiment(s), both PP and KR analyzed the results. PP wrote the paper. All authors reviewed the manuscript.

### Conflict of interest statement

The authors declare that the research was conducted in the absence of any commercial or financial relationships that could be construed as a potential conflict of interest.
