# Supplementary material for: Learning to Generate Sequences with Combination of Hebbian and Non-hebbian Plasticity in Recurrent Spiking Neural Networks
Source: Front Neurosci. 2017 Dec 12;11:693. doi: 10.3389/fnins.2017.00693 (PMC5733011; doi:10.3389/fnins.2017.00693)
Supplement: Supplementary file 1 [file DataSheet1.pdf]

Appendix: Learning to Generate Sequences with Combination of Hebbian and non-Hebbian Plasticity in Recurrent Spiking Neural Networks

Priyadarshini Panda* and Kaushik Roy
*Correspondence:
Priyadarshini Panda
pandap@purdue.edu

Neuron and Synapse Model

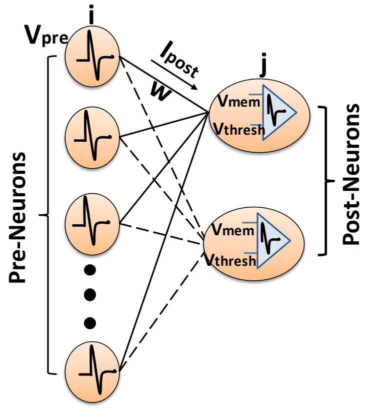

Figure S1. A typical SNN architecture consisting of pre-neurons and post-neurons interconnected by synapses. The pre-synaptic voltage spike V_{pre} is modulated by the synaptic weight, w, to get the resultant post-synaptic current, I_{post}. The post-neuron integrates the current from each interconnected pre-neuron that causes its membrane potential, V_{mem}, to increase and spikes when the potential crosses a certain threshold, V_{thresh}.

We use the standard Spiking Neural Network (SNN) dynamics to model the neuronal and synaptic changes in our reservoir spiking model. As shown in Fig. S1, a post-neuron receives the pre-synaptic spikes modulated by the synaptic strengths that results in a change in its membrane potential. Synapses are modeled by exponentially decaying conductance changes wherein the conductance increases by a synaptic weight, *w*, on arrival of a pre-synaptic spike. Otherwise, the conductance continues to decay. The dynamics of both Inhibitory (Inh) and Excitatory (Exc) conductance for corresponding pre-synaptic neurons are

$$\tau_e \frac{dg_e}{dt} = -g_e \;\; \tau_i \frac{dg_i}{dt} = -g_i \tag{A.1}$$

where $\tau_e(2ms), \tau_i(4ms)$ are the time constants for Excitatory and Inhibitory post-synaptic potentials.

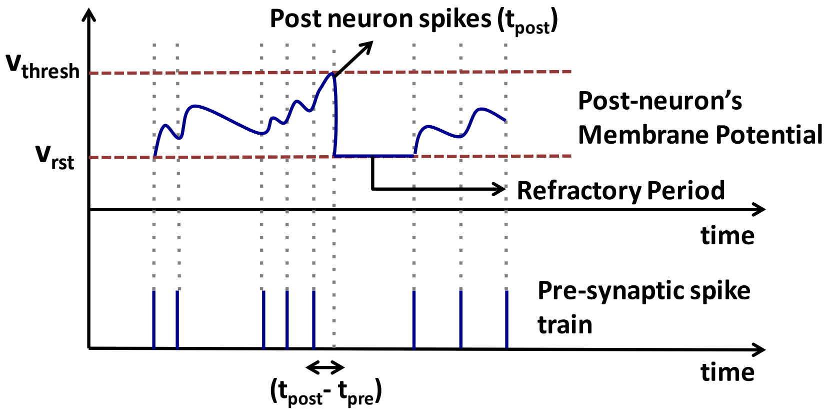

Figure S2. The Leaky-Integrate-and-Fire dynamics of the membrane potential of a post-neuron that increases upon the arrival of pre-synaptic spike and decays subsequently. The post-neuron fires when the potential exceeds the threshold V_{thresh}. The potential is then reset to V_{rst} and a refractory period ensues during which the neuron is prohibited from firing. The relative timing of the post-neuron and pre-neuron spikes ($t_{post} - t_{pre}$) determines the synaptic potentiation.

We use the Leaky-Integrate-and-Fire (LIF) model illustrated in Fig. S2 to simulate the membrane potential V_{mem} of a neuron as

$$\tau_{mem} \frac{dV_{mem}}{dt} = (V_{rest} - V_{mem}) + g_e(V_{exc} - V) + g_i(V_{inh} - V) \tag{A.2}$$

where V_{rest} is the resting membrane potential (-65 mV/-60 mV for Exc/Inh neurons, respectively), V_{exc} (0 mV) and V_{inh} (-100 mV) the equilibrium potentials of Exc and Inh synapses, τ_{mem} is the leak time constant (60 ms/ 10 ms for Exc/Inh neurons). The LIF model causes V_{mem} to increase or decrease when pre-synaptic spikes are received and to otherwise decay exponentially. The post-neuron fires when V_{mem} crosses the membrane threshold v_{thresh} (-52 mV/ -40 mV for Exc/Inh neurons, respectively) and then its membrane potential is reset to v_{rst} (-65 mV/ -45 mV for Exc/Inh neurons). After each firing event, a refractory period (5 ms/ 2 ms for Exc/Inh neurons) ensues during which the post-neuron is inhibited from firing even if additional input spikes arrive. Please note that both inhibitory and excitatory neurons in our proposed reservoir architecture follow the above LIF dynamics with different parameters. The homeostatic membrane threshold is evaluated as $v_{thresh} + \theta$, where θ is increased by 0.1 mV when the neuron fires and then decays exponentially with a very large time constant (10^8 ms) to ensure that the adaptive threshold holds its values through the entire simulation.

Input Encoding

The input images of characters from the Char74K dataset (that comprise the visual words of the dictionary) are presented to the reservoir model as Poisson based spike trains, with firing rates proportional to the pixel intensity value. Specifically, each pixel intensity is divided by 6 resulting in firing rates between 0 and 42.5 Hz for standard grayscale valued pixels in the 0-255 range. As mentioned in the main manuscript, each input is presented to the reservoir for 350 ms. If the excitatory neurons in the reservoir, for a given input image, fire less than 5 spikes within the 350ms presentation time, the input firing rate is then increased by 20 Hz and the image is presented to the network again for 350 ms. This procedure is repeated until the reservoir outputs at least 5 spikes for the given input.

Training, Assignment and Inference

The plastic synapses connecting the input to the reservoir excitatory (In→Exc) neurons and the E→E connections within the reservoir are trained using the combined Hebbian/non-Hebbian Plasticity mechanism as mentioned in the manuscript. By the end of the training phase, the In→Exc connections learn to encode the generalized representations of each of the characters in the visual word/sequence while the E→E connections learn the correlation between each character. After the training, we set the learning rate for all plastic connections to zero and fix the adaptive membrane threshold of each excitatory neuron in the reservoir (as obtained from homeostasis). Then, the training set is presented once again to the reservoir model. Now, each excitatory neuron is assigned a particular class (or character) for which it spiked the most during this presentation. The assignment phase is the only time when training labels are used. Otherwise, the training of synaptic connections is completely unsupervised (i.e. no use of class labels).

During testing, when the reservoir is presented with a test image of an individual character from the visual word sequence, the test input is predicted to belong to the class represented by the group of assigned neurons with the highest average spiking rate. In addition, as described in the manuscript, we also monitor the second highest spiking activity among the reservoir neurons to obtain the next character of the sequence. The next character predicted is then presented to the reservoir and this process is repeated until the entire sequence has been generated. We use the value of the difference between the top-2 spiking activity to gauge whether or not sequence generation is done, as described in Section 3 above.

Model Summary

Model Summary	
**Population**	Three: Excitatory(N_E), Inhibitory(N_I), External Input; $N_E = 4N_I$
**Connectivity**	Input→Reservoir (30%), Random Connectivity for E→E (40%), E→I (40%), I→E (50%), I→I (10%) **(Refer to Fig. 1(a) of manuscript)**
**Input**	Poisson spike train with varying firing rates based on input pixel intensity **(Refer to Input Encoding section in Appendix)**
**Neuron Model**	Leaky Integrate-and-fire, Homeostatic threshold, Fixed refractory period **(Refer to Eqn. A.2 in Appendix)**
**Synapse Model**	Exponentially decaying conductance changes **(Refer to Eqn. A.1 in Appendix)**
**Plasticity Model**	Combined Hebbian/non-Hebbian plasticity<br>Input to Reservoir Connections **(Refer to Eqn. 1 of manuscript)**<br>Exc→Exc connections within the reservoir **(Refer to Eqn. 2, 3 of manuscript)**

Figure S3. Tabular description of our proposed reservoir model

Fig. S3 summarizes the model parameters with references to all equations for the sake of clarity.
